# Organic‐Microbial Strategies for Iron and Zinc Biofortification in Orange‐Fleshed Sweet Potato [ *Ipomoea batatas* (L.) Lam.]

**DOI:** 10.1002/fsn3.72323

**Published:** 2026-09-03

**Authors:** Emmanuel Noumsi‐Foamouhoue, Paula Fernandes, Samuel Legros, Hassna Founoune‐Mboup, Bassirou Diallo, Komi Assigbetsé, Aboubacry Kane, Frédéric Feder, Jean‐Michel Médoc

**Affiliations:** ^1^ CIRAD, UPR Recyclage et Risque Montpellier France; ^2^ Laboratoire Mixte International IESOL, ISRA‐IRD Bel‐Air Center Dakar Senegal; ^3^ Recyclage et Risque, Université de Montpellier Montpellier France; ^4^ UPR HortSys, CIRAD Montpellier France; ^5^ HortSys, Université de Montpellier Montpellier France; ^6^ CIRAD, UPR Recyclage et Risque Saint‐Denis France; ^7^ Laboratoire National de Recherches Sur Les Productions Végétales (LNRPV), Institut Sénégalais de Recherche Agricole (ISRA) Dakar Senegal; ^8^ Institut de Recherche Pour le Développement (IRD), UMR Eco&Sols Montpellier France; ^9^ Département de Biologie Végétale FST Dakar Senegal; ^10^ Laboratoire Commun de Microbiologie (LCM), IRD‐ISRA‐UCAD Bel‐Air Center Dakar Senegal

**Keywords:** agronomic biofortification, beneficial microorganisms, mineral nutrition, orange‐fleshed sweet potato, organic inputs, sustainable agriculture

## Abstract

Orange‐fleshed sweet potato (OFSP) is widely promoted in sub‐Saharan Africa, including Senegal, for its contribution to vitamin A deficiency mitigation. However, its potential to improve mineral nutrition remains limited by low iron (Fe) and zinc (Zn) concentrations and their restricted bioavailability, which may contribute to persistent micronutrient deficiencies affecting 42% of children under 5 years and 37% of women of reproductive age in Senegal. In this context, organic waste products (OWPs) and microbial inocula (MIs) represent promising agroecological alternatives to conventional fertilizers for enhancing the nutritional quality of OFSP while valorizing locally available resources. This study aimed to evaluate the combined effects of various OWPs and MIs on tuber yield and Fe/Zn concentrations in OFSP within an agroecological production system. Field experiments were conducted across two contrasting seasons: a rainy season (June 2021–November 2021), followed by a dry season (December 2021–May 2022). The OFSP variety used was “Apomudem”. Two types of MIs were tested: local beneficial microorganisms (BMs), a bacterial‐fungal consortium derived from fermented forest litter, and mycorrhizal fungi (MF; *Glomus mosseae*). A factorial arrangement in a randomized complete block design with four replications was employed. Results revealed several combinations that significantly improved both yield and micronutrient content. The treatment combining poultry litter (PL) with BMs yielded 21 t ha^−1^ of tubers—a 2.6‐fold increase compared to the unfertilized control—and doubled Zn concentration to 20 mg kg^−1^ DM. Additionally, the combination of PL, BMs, and MF increased Fe concentration to 44 mg kg^−1^ DM, a 1.7‐fold increase. These effects varied with OWP and inoculum type, confirming that their interactions strongly influenced nutrient enrichment. Overall, combining OWPs with microbial inocula provides an effective agroecological approach for enriching crops with essential micronutrients and opens promising avenues for improving staple crop productivity and nutritional quality by effectively valorizing locally available resources, supporting agroecological transition, and food security.

## Graphical abstract

Orange‐fleshed sweet potato (OFSP) is valued for its provitamin A content but remains poor in iron (Fe) and zinc (Zn). In a two‐season field trial in Senegal, combining locally available organic waste products (poultry litter, PL; sewage sludge, SS) with microbial inocula (beneficial microorganisms, BM; mycorrhizal fungi, MF) significantly improved OFSP mineral nutrition. The most effective combination—PL+BM—increased tuber yield 2.6‐fold (21 t/ha) and doubled Zn concentration (20 mg/kg DM), while adding mycorrhizal fungi enhanced Fe concentration 1.7‐fold (44 mg/kg DM). This affordable, agroecological strategy offers a promising pathway to fight micronutrient deficiencies and strengthen food security in West Africa.

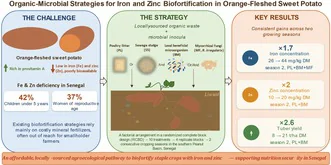

## 1 Introduction

Sweet potato ( *Ipomoea batatas* L.) is one of the most beneficial crops for human health (Amoanimaa‐Dede et al. 2019; Li et al. 2014). Globally, China is the leading producer, with an estimated 2.3 million hectares under cultivation and an annual output of approximately 52 million tonnes in 2023 (FAOSTAT 2025). In Senegal, sweet potato is grown on about 2500 ha, with a production of 15,000 t and an average yield of 45.5 t ha^−1^ in the same year (FAOSTAT 2025). Due to its high caloric density (123 cal/100 g) and rich content in carbohydrates, vitamins, and certain micronutrients, sweet potato plays a strategic role in community‐based nutrition improvement programs (Best et al. 2016; Guclu et al. 2023). Among the different varieties, orange‐fleshed sweet potato (OFSP), introduced and widely adopted in rural areas of Senegal, is notable for its high provitamin A content, with beta‐carotene levels ranging from 5500 to 11,030 μg/100 g of fresh weight (Abdou et al. 2018). These varieties have been integrated into several programs aimed at combating malnutrition, particularly in the Peanut Basin, to address vitamin A deficiencies and reduce poor dietary habits (Sylla et al. 2020; Thiam, Diouf, Médoc, et al. 2025).

However, despite their nutritional benefits, these varieties remain low in iron (Fe) (5 to 17 mg kg^−1^) and zinc (Zn) (1 to 8 mg kg^−1^), with concentrations insufficient to meet the daily nutritional requirements recommended by the European Food Safety Authority (EFSA), particularly for children aged 1–3 years (10 mg/day of Fe, 7 mg/day of Zn) and women of reproductive age (14 mg/day of Fe, 10 mg/day of Zn) (EFSA Panel on Dietetic Products, Nutrition and Allergies 2015, 2014). Furthermore, the bioavailability of these micronutrients is often limited due to the presence of antinutritional compounds, such as phytates (Koua et al. 2018). Enhancing the Fe and Zn content in OFSP tubers is essential to effectively combat micronutrient deficiencies. In Senegal, these deficiencies affect 42% of children aged below 5 years and 37% of women of reproductive age (COSFAM 2022).

Agronomic biofortification has emerged as a promising approach to improve crop micronutrient concentrations by enhancing nutrient availability and plant uptake through soil management and beneficial plant–microbe interactions (Bouis and Saltzman 2017; Frossard et al. 2000). Compared with genetic approaches or post‐harvest supplementation strategies, it relies on the optimization of nutrient inputs and biological processes occurring within the soil–plant system. Current agronomic approaches include the use of mineral fertilizers, organic inputs, and beneficial microorganisms to promote micronutrient acquisition and accumulation in edible plant tissues (Çakmak and Kutman 2018; Chattha et al. 2017; Kamran et al. 2017; Khan et al. 2019; McGrath et al. 2012).

Most existing research on sweet potato's Fe and Zn biofortification has focused on using mineral fertilizers applied to the soil or foliage (Abu Md Mostafizar Rahman et al. 2024; Sun et al. 2019; Tan et al. 2019). In contrast, studies exploring the use of organic materials and/or beneficial microorganisms remain scarce. Yet, although mineral fertilizers have proven effective in increasing micronutrient content in tubers (Sun et al. 2019; Tan et al. 2019; Zevallos et al. 2024; Zhang et al. 2022), their high cost and limited availability in rural areas hinder widespread adoption. Conversely, locally sourced organic wastes—animal manure, crop residues, agro‐industrial by‐products—are cheaper and widely accessible in rural Senegal, supported by national agroecological policies and community‐led initiatives (Maodo 2018; McClintock and Diop 2005). Moreover, excessive use of mineral fertilizers can degrade soil quality and contaminate groundwater (Pahalvi et al. 2021; Robertson and Vitousek 2009).

In this context, the combined use of organic inputs and beneficial microorganisms represents a promising strategy to improve Fe and Zn nutrition in OFSP while contributing to agroecological production systems. OWPs, originating from various human and agricultural activities, encompass a wide range of organic residues and by‐products, including materials of animal, plant, urban, industrial, and agro‐industrial origin (Carpio et al. 2021; Schievano et al. 2009; Sharma et al. 1997). Beyond their direct contribution of micronutrients and organic matter, OWPs can improve soil nutrient availability, whereas MIs may stimulate nutrient transformation, mobilization, and plant uptake. Among these inocula, BMs, produced through the anaerobic fermentation of forest litter collected from minimally disturbed areas, represent a locally adapted microbial resource containing diverse functional groups, including photosynthetic bacteria, lactic acid bacteria, actinomycetes, and fungi (Daly and Stewart 1999; Mahmud et al. 2021; Ney et al. 2020). However, forest litter harbors highly diverse microbial communities, including microorganisms whose functional attributes and biosafety profiles remain insufficiently characterized. During anaerobic fermentation, the production of organic acids lowers pH and promotes the enrichment of acid‐tolerant fermentative microorganisms while suppressing a substantial proportion of undesirable microbial populations (Marois et al. 2023). Nevertheless, the microbial composition of BMs remains strongly influenced by the source material and fermentation conditions, underscoring the need for microbial characterization to ensure their biosafety (Zoumman et al. 2025; Marois et al. 2023).

Previous research across different cropping systems has demonstrated that organic inputs and beneficial microorganisms can enhance micronutrient accumulation in edible plant tissues. For example, long‐term sewage sludge (SS) application in England increased wheat grain Zn concentration from 36 to 73 mg kg^−1^ (+103%) (McGrath et al. 2012). Similarly, inoculation with Zn‐solubilizing bacteria (*Rhizobium*, *Pseudomonas*, *Enterobacter*, and *Pantoea*) increased Zn concentration in wheat grain from 2.68 mg kg^−1^ in the control to 6.96 mg kg^−1^ (+160%) (Kamran et al. 2017). In addition, organic waste compost application under Spanish field conditions increased Fe concentration in potato tubers from approximately 35 to 115 mg kg^−1^ (+229%) (Escobedo‐Monge et al. 2020) reported that organic waste compost enhanced Fe accumulation in potato tubers under Spanish field conditions, with concentrations rising from approximately 35 mg kg^−1^ in the control to 115 mg kg^−1^ (+229%). Despite these encouraging results, evidence remains scarce regarding Fe and Zn biofortification of OFSP under tropical agroecological production systems in West Africa.

Based on this background, we formulated two key hypotheses: (i) OWPs, due to their high micronutrient content, can significantly increase Fe and Zn concentrations in OFSP tubers; and (ii) microbial inocula can enhance OWP mineralization, nutrient mobilization, and uptake by plants. Therefore, this study aimed to assess the combined effects of OWPs and microbial inocula on Fe and Zn concentrations in OFSP tubers within an agroecological framework.

## 2 Materials and Methods

### 2.1 Experimental Particulars

A field experiment was conducted during two successive cropping seasons corresponding to the local climatic regime: a rainy season (June–November 2021; 820 mm cumulative rainfall) and a subsequent dry season (December 2021–May 2022; no recorded rainfall) (Figure S1). The second season was established on the same experimental units to evaluate both the response to newly applied inputs and the residual influence of the first application. Therefore, maintaining an identical treatment structure across seasons enabled the assessment of cumulative and carry‐over effects of OWPs and MIs on OFSP performance. Rainfall during the rainy season was sufficient to meet crop water requirements, and no irrigation was applied. During the dry season, irrigation was provided throughout crop development to compensate for the absence of precipitation and avoid water limitation, ensuring comparable water availability for crop growth across seasons.

**FIGURE 1:**
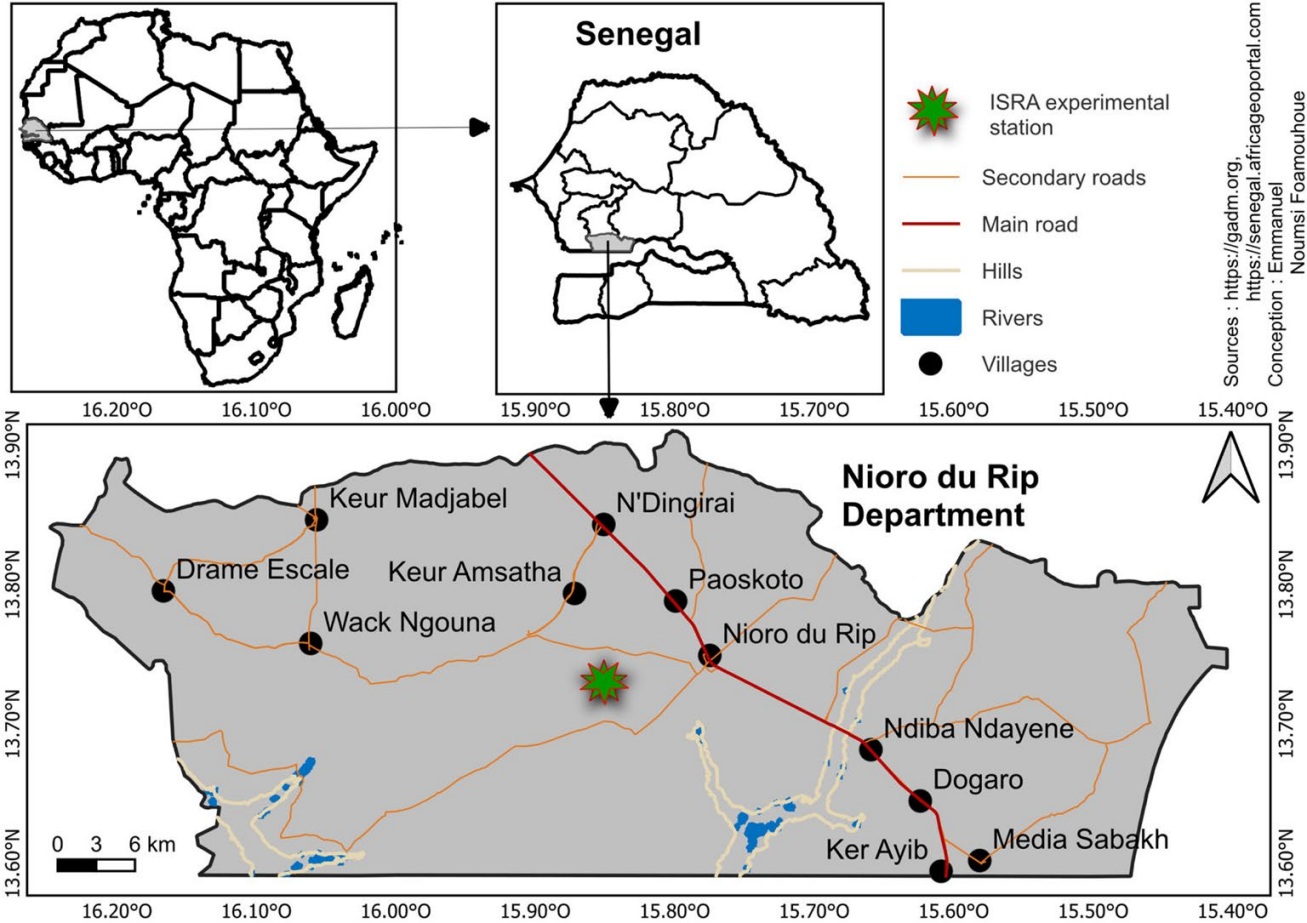
Map of the study site–Nioro du Rip, southern Peanut Basin, Senegal.

The field trial was established at the Institut Sénégalais de Recherches Agricoles (ISRA) experimental station in Nioro du Rip, south of the Peanut Basin (13°45′29″ N, 15°47′12″ W; 20–30 m above sea level; Figure 1). During the rainy season (June–November 2021), temperatures ranged from 28°C to 30°C, relative humidity from 50% to 75%. During the dry season (December 2021–May 2022), temperatures ranged from 27°C to 31°C, relative humidity from 20% to 55% (detailed meteorological data are shown in Figure S1). The soil was a Lixisol (Sall et al. 2016; IUSS Working Group WRB 2015), with low total organic carbon (2.8 g kg^−1^ DM), total organic nitrogen (0.20 g kg^−1^ DM), cation exchange capacity (1.47 cmol(+) kg^−1^ DM), and total Zn (< 50 mg kg^−1^ DM) levels (Table 1). The OFSP variety “Apomudem” cuttings, supplied by the ISRA seed service, were used in the experiment. MF and “Apomudem” were selected due to demonstrated effects on improving micronutrient bioavailability in OFSP biomass, as evidenced by greenhouse‐based bioavailability tests conducted within the OR4Food project (Alia 2021). This variety was also selected for its high yield, resistance to local diseases, high beta‐carotene content, and favorable acceptance by local farmers and consumers. The input treatments included two contrasting organic resources: PL, representing an agricultural residue, and SS, originating from an urban wastewater treatment process. Both materials were collected in the Dakar region. PL consisted of dried poultry droppings mixed with peanut shells collected from a poultry house in Sangalkam, whereas SS was obtained after wastewater treatment and open‐air drying at the Cambérène treatment station. Two types of microbial inocula were tested. The first microbial inoculum consisted of BMs, produced by fermenting forest litter collected in the Peanut Basin region with peanut shells according to the protocol described by Noumsi‐Foamouhoue et al. (2023). Second, an MF *Glomus mosseae* was provided by the Laboratoire Commun de Microbiologie (LCM, IRD‐ISRA‐UCAD). The inoculum of *Glomus mosseae* was produced through pot cultivation on sterile sand, using pearl millet ( *Pennisetum glaucum* ) as the host plant. The final product is a crude mixture consisting of sand, spores, mycelial hyphae, and fragments of colonized roots (Thioye et al. 2021). The main chemical properties of OWPs and BMs are presented in Table 1. The microbial composition of BMs is shown in Table S1. PL and SS were selected because they represent widely available organic resources with contrasting physicochemical properties and relevant Fe and Zn contents. Their potential nutrient release dynamics had previously been characterized under controlled incubation conditions (Noumsi‐Foamouhoue et al. 2023). BMs were selected based on the diversity of their associated microbial communities and their previously demonstrated capacity to stimulate organic matter mineralization and nutrient cycling (Noumsi‐Foamouhoue et al. 2023).

**TABLE 1:** Main soil, organic waste product (OWP), and beneficial microorganism (BM) properties.

	Soil	PL	SS	BM
TOC (g 100 g^−1^ DM)	0.28	27.36	23.75	34.24
TON (g kg^−1^ DM)	0.20	65	25.50	9.70
N‐NO_3_ (mg kg^−1^ DM)	0.80	n.d.	n.d.	n.d.
N‐NH_4_ (mg kg^−1^ DM)	5.40	n.d.	n.d.	n.d.
C/N	13	4.21	9.31	35.30
Total P (mg kg^−1^ DM)	65	12,941	9274	580
Assim. P (mg kg^−1^ DM)	7	1141	196	192
CEC (cmol(+) kg^−1^ DM)	1.47	n.d.	n.d.	n.d.
WC (g 100 g^−1^)	3.32	8.12	3.74	59
pH_(H2O)_	6.67	7.57	6.98	7.28
Fe (mg kg^−1^ DM)	4728	6210	15,724	4.34
Zn (mg kg^−1^ DM)	< 50	326	364	20
Sand (g 100 g^−1^ DM)	83.50	n.d.	n.d.	n.d.
Silt (g 100 g^−1^ DM)	9.50	n.d.	n.d.	n.d.
Clay (g 100 g^−1^ DM)	5.40	n.d.	n.d.	n.d.

*Note:* Analytical methods are described in Section 2.3.

Abbreviations: BM, local beneficial microorganisms; CEC, cation‐exchange capacity; DM, dry matter, 105°C; n.d., not determined; PL, poultry litter; SS, sewage sludge; TOC, total organic carbon; TON, total organic nitrogen; WC, water content.

### 2.2 Trial Establishment and Maintenance

The experiment followed a factorial arrangement in a randomized complete block design with four replicates. The experimental area measured 37 × 15 m (555 m^2^) and comprised 10 treatments (Table 2). Before treatment application, the field was prepared by disc plowing. Following soil pre‐irrigation, the OWPs were incorporated into the designated plots to a depth of approximately 20–30 cm. Application rates were 2.05 t DM ha^−1^ (2.23 t fresh matter ha^−1^) for PL and 7.27 t DM ha^−1^ (7.56 t fresh matter ha^−1^) for SS. Application rates were calculated from the N, P, and K requirements of sweet potato together with fertilizer equivalency coefficients (Gret et al. 2003; Leclerc 2001) (Table S2). Solid BM inoculum was incorporated into the designated plots at 9.44 t DM ha^−1^ (equivalent to 15 t crude matter ha^−1^). The quantities of Fe and Zn provided by OWPs and BMs are reported in Table S3. MF inoculum was placed directly into the planting holes at a rate of 1.88 t ha^−1^. OFSP vine cuttings were transplanted at a density of 38,095 plants ha^−1^, on June 10, 2021 (Season 1) and December 7, 2021 (Season 2). Each cutting was inserted obliquely, with approximately two‐thirds of its length buried in the soil. For the mineral biofortification treatment (Fe_Zn), foliar spray solutions containing FeSO_4_·7H_2_O (0.81 g L^−1^) and ZnSO_4_·7H_2_O (5 g L^−1^) were prepared. Two applications were performed at the early growth and tuberization stages, corresponding to cumulative application rates of 0.57 kg ha^−1^ FeSO_4_·7H_2_O and 3.5 kg ha^−1^ ZnSO_4_·7H_2_O (Chattha et al. 2017; Godsey et al. 2003). Chemical supplements (Table S2) were applied to the corresponding elementary plots at the early growth (phosphate fertilizer) and flowering (potassium fertilizer) stages. A liquid BM inoculum, produced by anaerobic fermentation of the solid inoculum for 15 days, was subsequently applied to foliage (2 mL m^−2^ every 7 days) and soil (10 mL m^−2^ every 14 days). The distance between elementary plots was 1 m. Within each plot, planting pockets were spaced 40 cm apart on the row and 1 m between rows. The 10 treatments (summarized in Table 2) consisted of combinations of OWPs (PL or SS) and microbial inocula (BMs and/or MF). The Control received no input, while the Fe_Zn treatment received foliar sprays of FeSO_4_·7H_2_O and ZnSO_4_·7H_2_O. The origin and description of each product are detailed in Section 2.1. Without rainfall, sprinkler irrigation was applied to the plot (Image S1). OFSP was harvested (Image S2) 5 months after transplanting the cuttings.

**TABLE 2:** Treatment factor modalities.

Treatments	Chemical fortification	OWP		MI	
		PL	SS	BM	MF
Control					
Fe_Zn	x				
PL		x			
SS			x		
PL_BM		x		x	
SS_BM			x	x	
PL_MF		x			x
SS_MF			x		x
PL_BM_MF		x		x	x
SS_BM_MF			x	x	x

Abbreviations: BM, local beneficial microorganisms; MF, mycorrhizal fungi; MI, microbial inocula; OWP, organic waste product; PL, poultry litter; SS, sewage sludge.

### 2.3 Sample Preparation and Analysis

After harvesting, the weights of OFSP tubers were assessed by weighing (0.01 g scale). OFSP tuber samples (washed, peeled, and oven‐dried at 65°C for 72 h) were ground using a ceramic grinder to avoid metal contamination. The powdered samples were stored in polypropylene pots and kept at room temperature (25°C ± 2°C). The analytical methods followed standard French and international procedures (AFNOR/ISO) for soil and plant analyses. Fe and Zn concentrations in tubers were determined by extraction with ultra‐pure 1 M HNO_3_ and quantified by microwave plasma atomic emission spectrometry (MP‐AES 4200, Agilent Technologies, Santa Clara, CA, USA). Total phosphorus was determined after wet digestion with HNO_3_ and H_2_O_2_, followed by continuous flow analysis using an AutoAnalyzer 3 (AA3; SEAL Analytical, Norderstedt, Germany) according to International Organization for Standardization (2018). Assimilable phosphorus was measured using the Murphy–Riley method (Murphy and Riley 1962). Total carbon and total nitrogen were determined by direct combustion using a Thermo Finnigan Flash EA 1112 elemental analyzer (Thermo Fisher Scientific, Waltham, MA, USA) according to International Organization for Standardization (1995). Nitrate nitrogen (NO_3_^−^–N) and ammonium nitrogen (NH_4_^+^–N) were extracted with 1 M KCl according to International Organization for Standardization (2005) and quantified by colorimetric analysis using an AutoAnalyzer 3 (AA3; SEAL Analytical, Norderstedt, Germany). Cation‐exchange capacity (CEC) was determined by extraction with 1 M KNO_3_, and the extracted cations were quantified using atomic absorption spectrometry (AA220 FS, Agilent Technologies, Santa Clara, CA, USA). Soil pH was measured in a soil‐to‐water suspension with a 1:5 ratio (International Organization for Standardization 2021), and the same method was applied to determine the pH of OWPs and BMs. Soil texture (sand, silt, and clay fractions) was assessed after organic matter removal with H_2_O_2_ and particle dispersion using Na_4_P_2_O_7_ (International Organization for Standardization 2020).

### 2.4 Calculation and Statistical Analysis

The amounts of Fe and Zn exported in OFSP tubers on an area basis (kg ha^−1^) were calculated using the following equation:

$$Y_{\mathrm{MN}\left(x\right)}\left[\mathrm{kg}\;{\mathrm{ha}}^{-1}\right]=\frac{C_{\mathrm{MN}\left(x\right)}\left[\mathrm{mg}\;{\mathrm{kg}}^{-1}\right]\times {\mathrm{DM}}_{\left(x\right)}\left[t\;{\mathrm{ha}}^{-1}\right]}{1000}.$$

where YMN(x) denotes the Fe or Zn yield in tuber sample x, CMN(x) is the corresponding micronutrient concentration, and DM(x) represents the sample dry matter.

All statistical analyses were conducted in R (version 4.4.3). Residual normality was assessed with the Shapiro–Wilk test, whereas homogeneity of variances was evaluated using Levene's test. The main effects of season and treatment and their interaction were evaluated using a two‐way analysis of variance (ANOVA). A non‐parametric alternative—the Scheirer–Ray–Hare test—was used for non‐normally distributed data to evaluate main and interaction effects. For the factor Season, which has only two levels, the *p*‐value of the main effect from the two‐way ANOVA (or from the Scheirer–Ray–Hare test for non–normal data) was directly used to assess the difference between Season 1 and Season 2. Multiple comparisons among the 10 treatments were performed within each season using the Newman–Keuls test for normally distributed data, and the Kruskal–Wallis test followed by Dunn's post hoc test with Benjamini–Hochberg correction for non‐normally distributed data. All tests were performed at the 5% significance level. Data distributions are illustrated as boxplots. The horizontal line within each box represents the median, the box boundaries correspond to the first (Q1) and third (Q3) quartiles, and whiskers extend to observations located within 1.5 × IQR. Values beyond this interval are shown as outliers and were omitted from the graphical display.

## 3 Results

### 3.1 OFSP Yields

**FIGURE 2:**
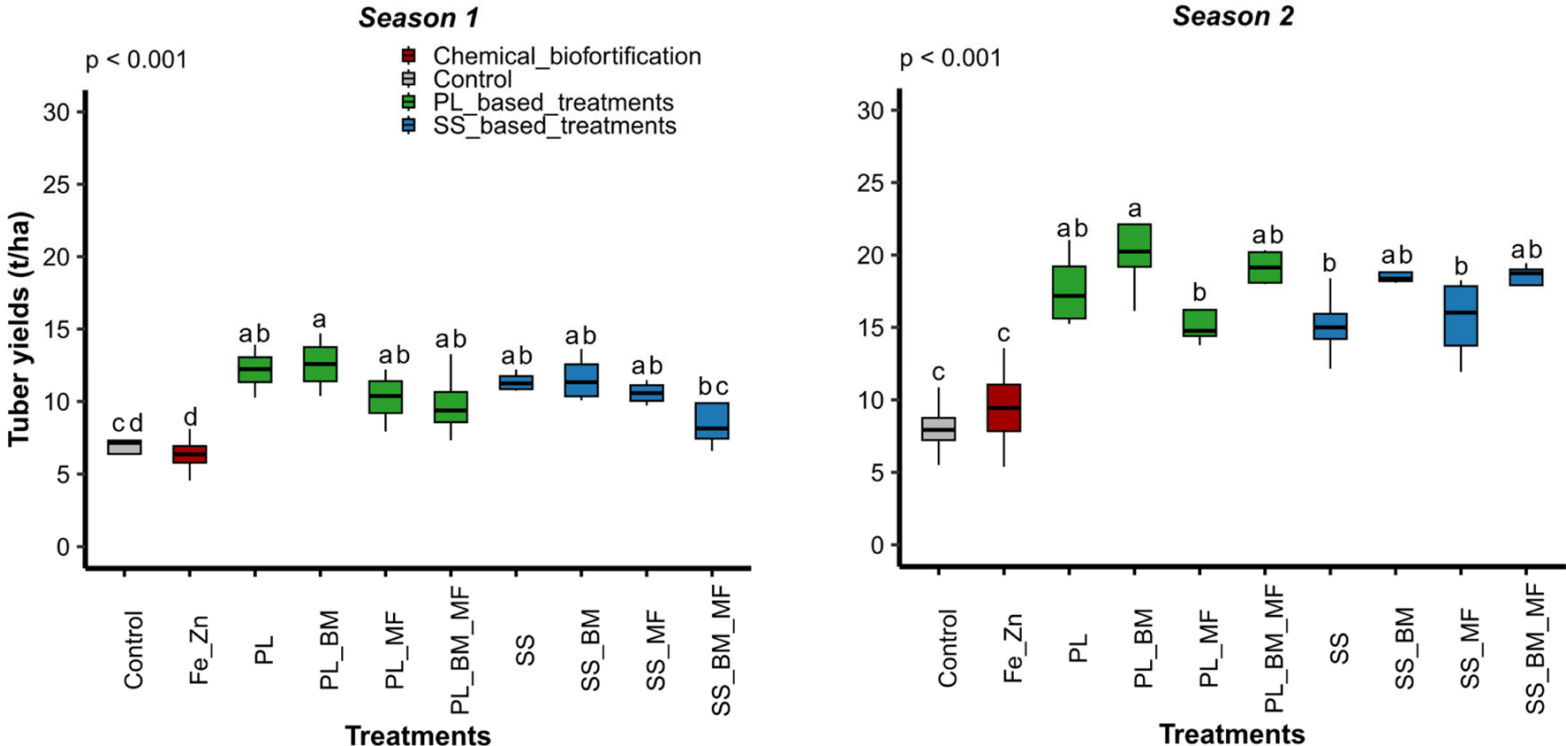
Tuber yields in Season 1 (June 2021–November 2021) and Season 2 (December 2021–May 2022) for treatments: Control (soil without input), Fe_Zn (mineral biofortification with FeSO_4_·7H_2_O + ZnSO_4_·7H_2_O), PL (poultry litter), PL_BM (poultry litter + local beneficial microorganisms), PL_MF (poultry litter + mycorrhizal fungi), PL_BM_MF (poultry litter + local beneficial microorganisms + mycorrhizal fungi), SS (sewage sludge), SS_BM (poultry litter + local beneficial microorganisms), SS_MF (poultry litter + mycorrhizal fungi), SS_BM_MF (poultry litter + local beneficial microorganisms + mycorrhizal fungi). Groups with different letters have significantly different means at *p* < 0.001.

The significant interaction between seasons and treatments emphasized the importance of comparing the efficacy of treatments within each season (Figure 2; Table S4).

Tuber yields are significantly higher in Season 2 than in Season 1.

Tuber yields are significantly higher (*p* < 0.001) in all the treatments involving an OWP (mean range: 9.19–12.56 t ha^−1^ in Season 1 and 15.13–21.06 t ha^−1^ in Season 2) than control (mean: 6.55 t ha^−1^ in Season 1 and 8.05 t ha^−1^ in Season 2) or Fe_Zn (mean: 6.35 t ha^−1^ in Season 1 and 9.45 t ha^−1^ in Season 2). The exception is SS_BM_MF, which is not significantly different from the control in Season 1.

The most important result is the effect of the combination of PL and BMs on the tuber yields. PL_BM exhibited the highest tuber yields, particularly during Season 2, where its yield (21.06 ± 4.8 t ha^−1^) was significantly higher (*p* < 0.001) than those of the treatments PL_MF (15.85 ± 2.89 t ha^−1^), SS_MF (15.55 ± 2.97 t ha^−1^), and SS (15.13 ± 2.55 t ha^−1^).

### 3.2 Fe and Zn Concentrations

**FIGURE 3:**
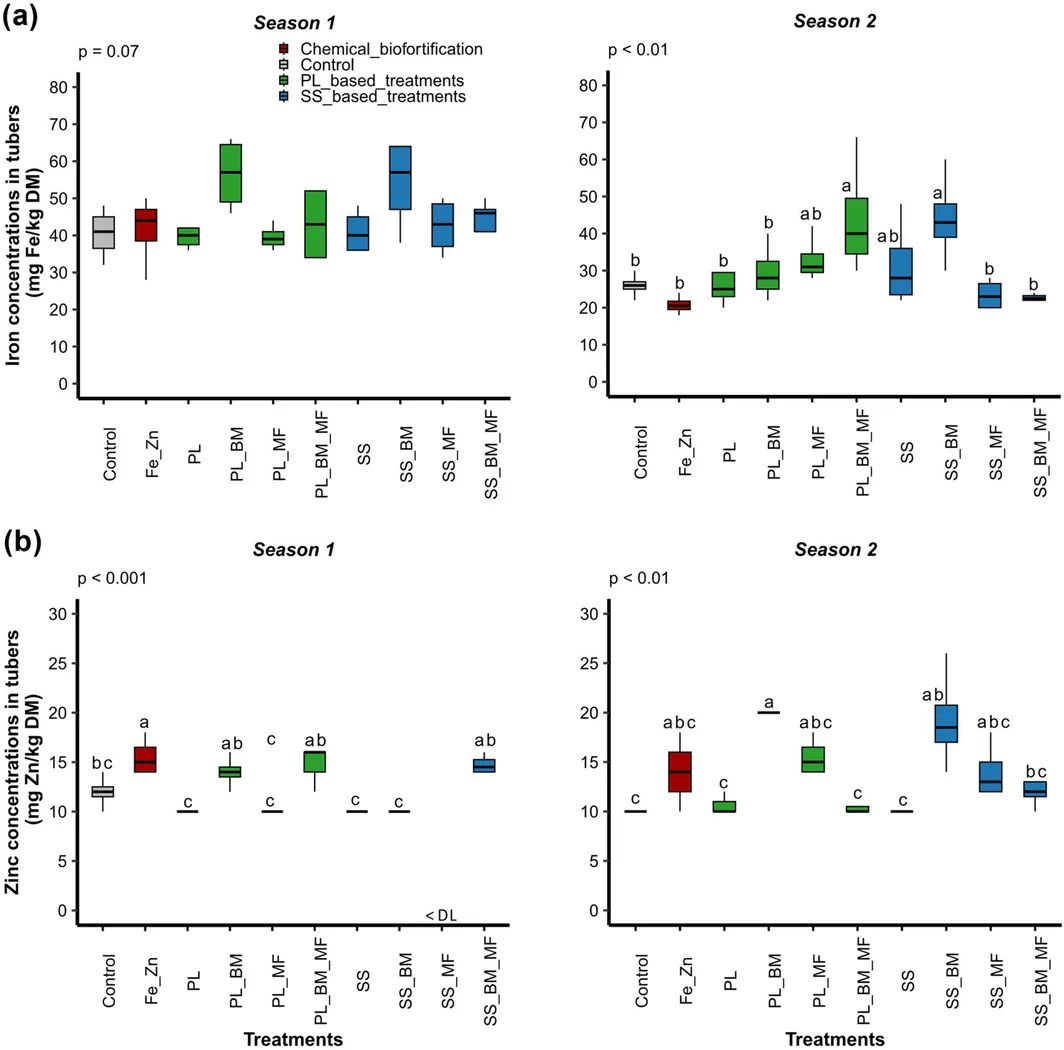
Iron (a) and zinc (b) concentrations in tubers in Season 1 (June 2021–November 2021) and Season 2 (December 2021—May 2022) for treatments: Control (soil without input), Fe_Zn (mineral biofortification FeSO_4_.7H_2_O + ZnSO_4_.7H_2_O), PL (poultry litter), PL_BM (poultry litter + local beneficial microorganisms), PL_MF (poultry litter + mycorrhizal fungi), PL_BM_MF (poultry litter + local beneficial microorganisms + mycorrhizal fungi), SS (sewage sludge), SS_BM (poultry litter + local beneficial microorganisms), SS_MF (poultry litter + mycorrhizal fungi), SS_BM_MF (poultry litter + local beneficial microorganisms + mycorrhizal fungi), DL (detection limit of the analysis spectrometer (10 ppm)). Groups with different letters have significantly different means at *p* < 0.01.

The two factors, treatments and transplanting seasons, significantly influenced Fe concentrations in tubers (Table S4). Fe concentrations in tubers are significantly higher in Season 1. Despite the non‐significant interaction between seasons and treatments, data were analyzed separately by season to better illustrate year‐to‐year variability in treatment responses (Figure 3a).

In Season 1, a trend toward higher Fe concentrations in tubers (*p* = 0.07) was observed with the PL_BM (56.5 ± 9.98 mg kg^−1^ DM) and SS_BM (54 ± 12 mg kg^−1^ DM) treatments, compared to all other treatments (mean range: 39.5–43 mg kg^−1^ DM). In Season 2, Fe concentrations in tubers of PL_BM_MF (44 ± 15.74 mg kg^−1^ DM) and SS_BM (44 ± 12.33 mg kg^−1^ DM) were significantly higher (*p* < 0.01) than those recorded in all other treatments (mean range: 20.75–29.5 mg kg^−1^ DM), except for PL_MF and SS, whose concentrations were not statistically different from those of PL_BM_MF and SS_BM.

In Season 1, Zn concentrations in tubers from the Fe_Zn treatment (15.5 ± 1.91 mg kg^−1^ DM) were significantly higher (*p* < 0.001) than those observed in the control (12 ± 1.63 mg kg^−1^ DM) and other treatments with similar Zn concentrations (10 ± 0 mg kg^−1^ DM), namely PL, PL_MF, SS, SS_BM, and SS_MF. Furthermore, no significant differences were observed between Fe_Zn and the treatments PL_BM (14 ± 1.63 mg kg^−1^ DM), PL_BM_MF (14.66 ± 2.3 mg kg^−1^ DM), and SS_BM_MF (14.75 ± 0.96 mg kg^−1^ DM).

In Season 1, PL_BM (14 ± 1.63 mg kg^−1^ DM) and PL_BM_MF (14.67 ± 2.31 mg kg^−1^ DM) had significantly higher Zn concentrations (*p* < 0.001) than the other PL‐based treatments (PL and PL_MF), both of which showed identical values (10 ± 0 mg kg^−1^ DM). Similarly, SS_BM_MF (14.75 ± 0.96 mg kg^−1^ DM) had significantly higher concentrations (*p* < 0.001) than SS and SS_BM (10 ± 0 mg kg^−1^ DM). In Season 2, PL_BM showed significantly higher Zn concentrations in tubers (20 ± 0 mg kg^−1^ DM; *p* < 0.01) than the control (10 ± 0 mg kg^−1^ DM), PL (10.67 ± 1.15 mg kg^−1^ DM), and PL_BM_MF (10.5 ± 1 mg kg^−1^ DM). Similarly, SS_BM (19.25 ± 4.99 mg kg^−1^ DM) had significantly higher Zn concentrations (*p* < 0.01) than SS (10 ± 0 mg kg^−1^ DM).

### 3.3 Fe and Zn Yields

**FIGURE 4:**
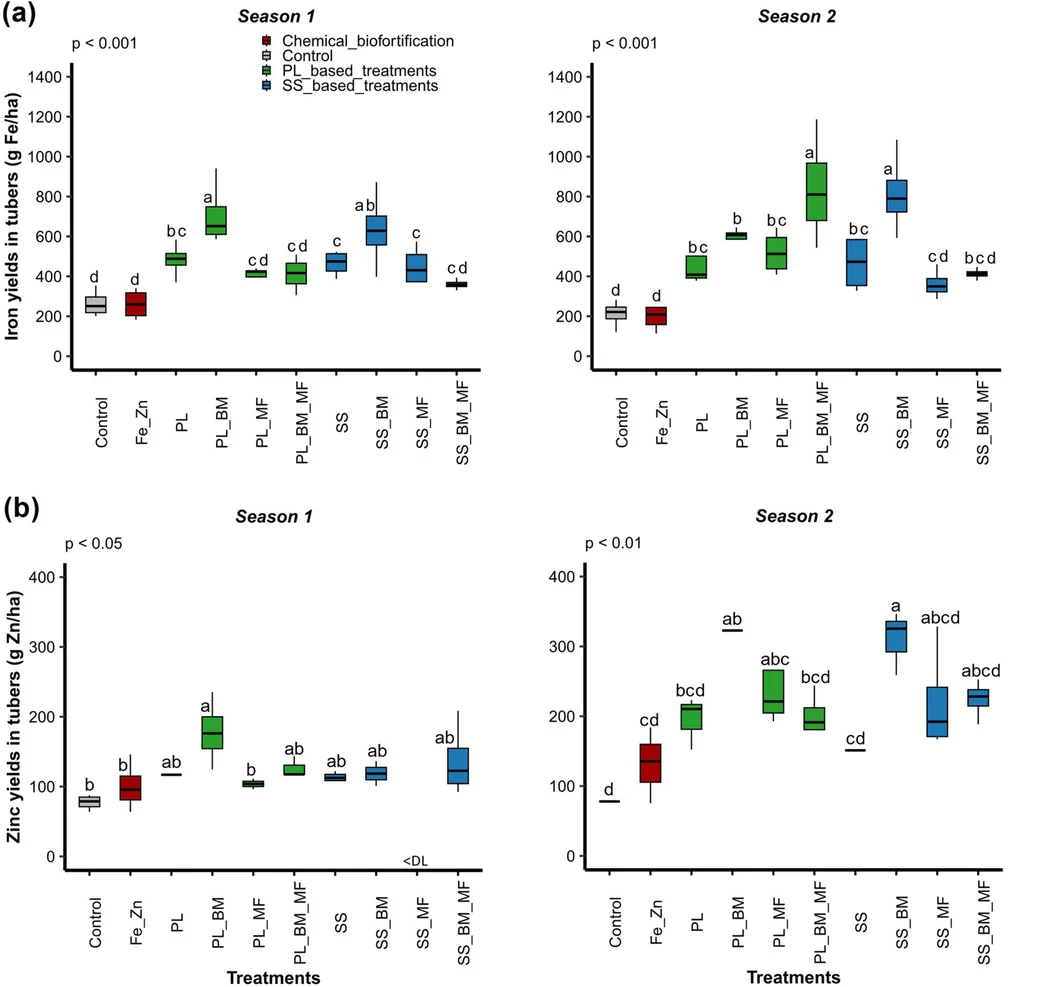
Iron (a) and zinc (b) yields in tubers in Season 1 (June 2021–November 2021) and Season 2 (December 2021–May 2022) for treatments: Control (soil without input), Fe_Zn (mineral biofortification FeSO_4_.7H_2_O + ZnSO_4_.7H_2_O), PL (poultry litter), PL_BM (poultry litter + local beneficial microorganisms), PL_MF (poultry litter + mycorrhizal fungi), PL_BM_MF (poultry litter + local beneficial microorganisms + mycorrhizal fungi), SS (sewage sludge), SS_BM (poultry litter + local beneficial microorganisms), SS_MF (poultry litter + mycorrhizal fungi), SS_BM_MF (poultry litter + local beneficial microorganisms + mycorrhizal fungi), DL (detection limit of the analysis spectrometer (10 ppm)). Groups with different letters have significantly different means at *p* < 0.05.

Although there was no significant difference between the two seasons for Fe yields in tubers, the significant interaction between seasons and treatments showed that the effect of treatments on Fe yields in tubers depended on the transplanting season (Figure 4a; Table S4).

The Fe yields in tubers from most treatments involving an OWP (mean range: 451–707 g ha^−1^ in Season 1 and 465–837 g ha^−1^ in Season 2) were nearly a 2‐fold increase relative to the control (mean: 264 g ha^−1^ in Season 1 and 212 g ha^−1^ in Season 2) and Fe_Zn (mean: 261 g ha^−1^ in Season 1 and 193 g ha^−1^ in Season 2). Treatments (i) PL_MF, PL_BM_MF, and SS_BM_MF in Season 1, (ii) SS_MF and SS_BM_MF in Season 2, were not significantly different from the control and Fe_Zn treatments.

In Season 1, PL_BM had significantly higher Fe yields (707 ± 161 g ha^−1^; *p* < 0.001) than all other treatments involving PL (mean range: 400–482 g ha^−1^). Similarly, SS_BM had significantly higher Fe yields (631 ± 194 g ha^−1^; *p* < 0.001) than all other treatments involving SS (mean range: 360–464 g ha^−1^). In Season 2, PL_BM_MF (837 ± 273 g ha^−1^) and SS_BM (814 ± 203 g ha^−1^) had significantly higher Fe yields (*p* < 0.001) than all other treatments involving an OWP (mean range: 362–597 g ha^−1^).

The significant interaction between seasons and treatments highlighted the need to compare the efficacy of treatments within each season (Figure 4b; Table S4).

In Season 1, PL_BM showed significantly higher Zn yields (178 ± 46 g ha^−1^; *p* < 0.05) than the control (77 ± 10 g ha^−1^), Fe_Zn (100 ± 35 g ha^−1^), and PL_MF (104 ± 11 g ha^−1^). In Season 2, PL_BM (323 ± 0 g ha^−1^) had significantly higher Zn yields (*p* < 0.01) than the control (78 ± 0 g ha^−1^), Fe_Zn (132 ± 54 g ha^−1^), and SS (151 ± 0 g ha^−1^). SS_BM (362 ± 109 g ha^−1^) also exhibited significantly higher Zn yields (*p* < 0.01) than the control, Fe_Zn, SS, PL (195 ± 38 g ha^−1^), and PL_BM_MF (202 ± 30 g ha^−1^). Moreover, no significant difference was observed between PL_BM and SS_BM, which recorded the highest Zn yields in tubers, especially during Season 2.

## 4 Discussion

### 4.1 OWPs and Microbial Inocula Impacted OFSP Yields

The increase in tuber yields of OFSP observed in our study following the application of OWPs—up to +125% with the PL treatment compared to the control (Figure 2)—is consistent with results reported in the literature across diverse pedoclimatic contexts, as well as various types and doses of OWPs (Adekiya et al. 2022; Fernandes et al. 2021; Jaime‐Rodríguez et al. 2025; Navarro et al. 2020). For example, Oliveira et al. (2010) demonstrated a significant increase in total sweet potato root productivity on Regosol fertilized with cattle manure compared to the unfertilized control. The mechanisms underlying this yield response are well‐established in the literature on organic soil amendments: the addition of OWPs to sandy Lixisol soils—such as those at the Nioro du Rip experimental station—improves the cation exchange capacity, increases water‐holding capacity, and releases nitrogen and phosphorus progressively through mineralization, thereby creating more favorable rooting conditions for a storage‐root crop such as OFSP (Howe et al. 2024; Gomgnimbou et al. 2019; Luciens et al. 2014). Moreover, the tuber weight obtained with the PL treatment (18 t ha^−1^) is slightly higher than the results reported by Oliveira et al. (2010) with cattle manure application (17 t ha^−1^). This yield difference could be attributed to soil type and the nature and dose of fertilizers used. In our study, poultry litter with a C/N ratio of 4 (Table 1) was applied at 2 t ha^−1^ on a Lixisol, whereas Oliveira et al. (2010) applied cattle manure with a C/N ratio of 14 at 31 t ha^−1^ on a Regosol.

The PL_BM treatment recorded the highest tuber yield, reaching 21 t ha^−1^, representing an increase of 16% compared to PL, 32% compared to PL_MF, and 39% compared to SS (Figure 2). This result reflects the beneficial effect of BMs on plant growth and development (Bünemann et al. 2006; Dos Santos Sousa et al. 2020; Lelamo 2025; Pandey et al. 2025). Similarly, Younas et al. (2022) highlighted a stimulating effect of effective microorganisms on sweet potato yields, especially during the dry season. Several functional properties of the BM community (Table S1) likely underpin this yield advantage in OFSP. The dominance of *Lactobacillus* (40%), *Clostridium* (8%), and diverse *Firmicutes* within the consortium supports enhanced mineralization of the organic carbon and nitrogen fraction of OWPs, which is critical for meeting the high nitrogen demand of OFSP during the early vegetative phase. Phosphorus‐solubilizing genera (*Pantoea*, *Pseudomonas*, *Glomus*) further improve the plant‐available P pool—particularly relevant in the low‐P Lixisol of the study site (Table 1)—while the hyphal network of *Glomus* directly expands the root absorption surface of OFSP plantlets (Piliarová et al. 2019; Debasis et al. 2019). The additional effects of phytohormone producers (*Bacillus*, *Paenibacillus*) and antimicrobial genera (*Lactobacillus*, *Pseudomonas*) may have helped buffer OFSP plantlets against soil‐borne stresses during establishment (Gondal et al. 2021; Ling et al. 2022; Wang et al. 2021). Furthermore, the organic matter supplied by BMs (Table 1) could also improve plant nutrient availability.

The highest OFSP tuber yields were observed during Season 2, with an increase of 58% compared to that in Season 1 (Figure 2; Table S4). This notable improvement could be attributed to the combined effect of OWP residues and microbial inocula applied during Season 1, along with new OWP and microbial inocula in Season 2. The reapplication of OWPs and MIs in Season 2, combined with residual effects from Season 1, produced a soil environment progressively richer in labile nutrients and active microbial communities. This cumulative soil enrichment effect has been specifically documented for PL in tropical cropping systems, where carryover nitrogen and phosphorus pools contribute meaningfully to subsequent crop cycles (Freitas et al. 2025; Olsen et al. 2015). For OFSP—a crop that builds its storage organ over a 5‐month cycle—such progressive improvements in nutrient availability throughout the growing season are particularly consequential for final tuber yield. The highest tuber yields (21 ± 5 t ha^−1^) were obtained with the PL_BM treatment in Season 2, whereas the lowest yields (6 ± 1 t ha^−1^) were recorded with the Fe_Zn treatment in Season 1. These values differ from those reported by the Institut Sénégalais de Recherches Agricoles (ISRA) for the same variety (Apomudem), which are 5 t ha^−1^ under mineral fertilization and 3 t ha^−1^ without fertilization. They also diverge from the variety's potential yield, estimated at 10 t ha^−1^ (ISRA/URCI). Several factors can explain these discrepancies, notably the nature, composition, and dosage of the fertilizers applied. They may also result from environmental conditions specific to each trial, particularly the physicochemical and biological characteristics of the soils. These factors directly influence nutrient availability, microbial activity, and consequently, the agronomic outcomes of the crops.

These yield performance results are corroborated by the results of a parallel study conducted at the same experimental site in Nioro du Rip, under identical treatment conditions, where cowpea ( *Vigna unguiculata* ) showed increases in grain yield of up to 2.4‐fold and increases in haulm yield of up to 3.2‐fold when OWP applications were combined with microbial inocula (Noumsi‐Foamouhoue et al. 2026). The convergence of results between two contrasting species—a crop with an underground storage root and a grain legume—grown simultaneously on the same Lixisol confirms the robustness of the OWP × microbial inocula synergy under the semi‐arid soil and climate conditions of Senegal's Peanut Basin. The consistency of these responses across species reinforces the agronomic recommendation to co‐apply OWP with beneficial local microorganisms as a viable strategy to improve both the productivity and nutritional quality of staple crops in Senegal.

### 4.2 OWPs and Microbial Inocula Impact Fe Uptake

OWPs associated with microbial inocula, in some cases, significantly influenced the Fe concentrations and yields in the tubers compared to Fe_Zn and Control. We observed (i) a significant increase in Fe concentrations in tubers, reaching up to +69% (Figure 3a), and (ii) a notable increase in Fe yields in tubers, up to +295%, with PL_BM_MF compared to Fe_Zn and Control (Figure 4a).

Most available studies in the literature on Fe and Zn biofortification of sweet potato focus on foliar or soil application of synthetic fertilizers rich in Fe and Zn (Abu Md Mostafizar Rahman et al. 2024; Sun et al. 2019; Tan et al. 2019), while research on the use of organic materials and/or beneficial microorganisms remains very limited. In our study, no significant differences were observed in Fe concentrations and Fe yields in the tubers for the Fe_Zn treatment (foliar application of synthetic fertilizer) compared to the Control. This result contrasts with Sun et al. (2019), who reported a significant increase in Fe concentration in sweet potato tubers following foliar applications compared to an unfertilized control. This discrepancy could be explained by differences in the concentration of the applied solution and the number of applications. Indeed, in our protocol, two foliar sprays were performed—at early growth and tuber initiation stages—using a solution containing FeSO_4_·7H_2_O at 0.81 g L^−1^. In comparison, Sun et al. (2019) applied three sprays spaced 7 days apart with a more concentrated 1 g L^−1^ FeSO_4_·7H_2_O solution.

Two main mechanisms can explain the synergy observed with OWPs and microbial inocula in Fe uptake. The direct supply of Fe via OWPs—PL brought 25.46 kg Fe ha^−1^ and SS up to 228 kg Fe ha^−1^ (Table S5)—represents the first key mechanism. Beyond this quantitative input, organic amendments substantially modify soil Fe chemistry: by increasing the proportion of exchangeable and organically complexed Fe fractions, which are the forms most accessible to plant roots Shuman (1988), and by moderating soil pH and ionic strength in ways that shift the Fe^2+^/Fe^3+^ equilibrium toward the plant‐available ferrous form (Escobedo‐Monge et al. 2020). These physicochemical effects are particularly relevant in the slightly alkaline Lixisol of Nioro du Rip (pH 6.67, Table 1), where Fe solubility is inherently constrained. A second mechanism operates at the rhizosphere level through the BM community. The over‐mineralization of OWP carbon (+41%) recorded in incubation experiments with these BMs (Noumsi‐Foamouhoue et al. 2023) translates into enhanced microbial metabolic activity in the OFSP rhizosphere, which is known to increase Fe solubilization through rhizosphere acidification and exudate production (Dotaniya and Meena 2015). Within the BM consortium, *Clostridium* sp. (Table S1) specifically converts Fe^3+^ to the plant‐available Fe^2+^ form through reductive dissolution and organic acid secretion (Francis and Dodge 1991)—an ability of particular relevance in the Lixisol where Fe is predominantly present in oxidized, poorly soluble forms. Fe‐siderophore chelation by *Pseudomonas* and *Bacillus* (Table S1) further contributes by creating soluble Fe‐complex forms directly assimilable by OFSP roots plants (Khan et al. 2019; Wang et al. 1993). The additional contribution of *Glomus mosseae* (MF) to Fe accumulation in OFSP tubers—evidenced by the +50% Fe concentration in PL_BM_MF versus PL_BM in Season 2 (Figure 3a)—aligns with the documented capacity of arbuscular mycorrhizal fungi to directly access Fe‐rich soil microsites via extraradical hyphae, concentrating Fe in colonized root cortex cells before transferring it to the host plant (George et al. 1994). This hyphal Fe‐loading mechanism operates independently of root surface area expansion and may be especially effective in OFSP, whose fibrous root system provides extensive colonization sites. This dual effect of organic inputs and microbial inocula on Fe availability is fully consistent with the mechanistic framework recently summarized by Moreno‐Jiménez et al. (2024), who demonstrated, across various crops and soil types, that AMF modulate the expression of plant Fe transporter genes and reshape the rhizosphere microbiome, thereby collectively controlling Fe accumulation in plants. Their work further highlights that the greatest gains in micronutrients are achieved when AMF inoculation is combined with organic substrates that stimulate fungal hyphal development—which corresponds precisely to the strategy implemented in the PL_BM_MF combination of the present study.

In the present study, Fe concentrations in the tubers decreased by approximately 32% in Season 2, while tuber yields increased by 58% compared to that in Season 1 (Table S4). This is a well‐documented phenomenon in plant physiology: the dilution of mineral concentrations as plant organs expand. This ‘growth dilution’ has been formally described through mineral dilution curves in high‐yielding crops such as sugarcane, where N, P, and K concentrations in leaves declined as dry biomass increased Cruz and Guillaume (1999), and where Mn concentrations in stalks showed a similar inverse relationship with yield (Pouzet et al. 1999). That Zn did not follow this pattern in our study (Table S4) further underscores that Fe and Zn respond differently to the OWP × MI × season interactions.

An accumulation of Fe in the soil, resulting from repeated applications of Fe‐rich OWPs, was observed. This accumulation is notably illustrated by the significant gap between the total amounts of Fe applied and those extracted by the tubers (Table S5). Thus, Fe inputs via PL and SS were 25.46 and 228 kg ha^−1^, respectively. In comparison, Fe extractions by the tubers were 17.76 kg ha^−1^ (i.e., 69.76% of the input) for PL, and only 15.84 kg ha^−1^ (6.93%) for SS; this implies that the SS amendment leaves a much larger residual quantity of Fe in the soil (213 kg ha^−1^), compared to PL (7.7 kg ha^−1^). The large residual Fe pool left by SS applications (approximately 213 kg Fe ha^−1^ unextracted by OFSP tubers) raises environmental sustainability concerns specific to this double‐cropping system. In water‐saturated conditions, excessive soil Fe concentrations (> 800 ppm) may induce Fe^2+^ phytotoxicity in subsequent OFSP crops, impairing root function and reducing the uptake of competing cations including Zn^2+^ and K^+^ (Hasnine et al. 2017; Sahrawat 2005). This argues for preferring PL over SS in repeated applications, or for implementing controlled SS doses with soil Fe monitoring between crop cycles.

### 4.3 OWPs and Microbial Inocula Impact Zinc Uptake

OWPs associated with microbial inocula, in several cases, significantly influenced Zn concentrations and yields in the tubers compared to the control treatment. We observed (i) a significant increase in Zn concentrations in tubers, reaching up to +100% with PL_BM (Figure 3b), and (ii) a significant increase in Zn yields in tubers, up to +295% with SS_BM, compared to Fe_Zn and Control (Figure 4b).

The Fe_Zn treatment (foliar spraying of a synthetic fertilizer) led to a significant increase in Zn concentration in the tubers by +29% compared to the control. This result is consistent with findings reported in several studies (Abu Md Mostafizar Rahman et al. 2024; Tan et al. 2019; Laurie et al. 2015). However, the observed gain remains lower than that reported by Sun et al. (2019), who observed an increase of +59% with foliar spraying of ZnSO_4_·7H_2_O at 5 g L^−1^—the same concentration used in the present study. This difference could be explained by several factors, including the cultivated variety and its absorption potential, the number of applications, the plant developmental stage at the time of spraying, and pedoclimatic conditions that may influence the efficiency of foliar Zn absorption.

As with Fe, the synergy observed between OWPs and microbial inocula on Zn uptake can be explained by two main mechanisms. The first relates to the properties of OWPs. Adding organic materials rich in Zn (Table S3) helps improve the availability of this micronutrient in the soil. In the low‐CEC Lixisol of Nioro du Rip (CEC = 1.47 cmol(+) kg^−1^, Table 1), the improvement in Zn availability through OWP application has a dual basis. Organometallic complex formation releases Zn^2+^ ions into the soil solution at rates accessible to plant roots (Hafeez et al. 2013), while the parallel increase in soil organic matter elevates the CEC, retaining mobile Zn^2+^ in the rhizosphere rather than allowing leaching below the root zone. These two mechanisms are especially synergistic in coarse‐textured soils (83.5% sand, Table 1) where Zn retention is otherwise minimal (Carey et al. 2025; Campdelacreu Rocabruna et al. 2024). The second mechanism is based on the beneficial effects of MIs. Within the BM community, Zn mobilization is driven by a combination of enzymatic and chelation‐based pathways. *Pantoea*, present at 0.13% OTU (Table S1), was specifically shown to enhance root Zn uptake in rice through direct solubilization activity (Prajapati et al. 2021), while *Sphingobacterium*, *Pseudomonas*, and *Bacillus* act synergistically with soil organic matter to increase Zn phytoavailability in plants (Khalid and Ahmed 2022; Kamran et al. 2017). For OFSP—a tuberous crop with a highly developed fibrous root system—the loading of Zn into the storage organ further requires active transport across the root‐to‐tuber axis. The capacity of *Enterobacter* and *Pseudomonas* (Table S1) to upregulate ZIP‐family Zn transporter gene expression at the root level (Singh and Prasanna 2020) may thus be a particularly relevant mechanism for the tuber enrichment observed in the PL_BM and SS_BM treatments. Finally, the positive effect of mycorrhizae (MF) on Zn uptake in the established system is illustrated by the significant +48% increase in Zn observed with the SS_BM_MF treatment compared to SS_BM in Season 1. This result aligns with findings from Founoune‐Mboup et al. (2024), who reported a significant increase in Zn and Fe concentrations in millet biomass following inoculation with *Glomus mosseae*. Furthermore, Watts‐Williams and Cavagnaro (2018) showed that mycorrhizae induce the overexpression of genes encoding Zn transporters in barley roots, thereby contributing to improved micronutrient uptake. This molecular basis for the increase in Zn content induced by AMF is also supported by the meta‐analysis by Moreno‐Jiménez et al. (2024), who demonstrated that AMF consistently upregulate genes encoding Zn transporters of the ZIP family in colonized roots across multiple crop species and soil types, with the effects being particularly pronounced when mycorrhizal inoculation is combined with organic substrates that stimulate fungal mycelium development. These results provide a direct mechanistic explanation for the superior performance of the SS_BM_MF combination compared to SS_BM alone in terms of Zn accumulation in OFSP tubers observed during Season 1 of the present study.

While the overall average Zn concentration in OFSP tubers remained stable across the two seasons despite a 58% increase in tuber yield (Table S4), the significant season × treatment interaction indicates that treatment responses depended on the cropping season. This overall stability distinguishes Zn from Fe and points to a different regulatory balance in the source‐sink dynamics of this micronutrient. While Fe concentration decreased by 32% in Season 2 (Table S4), suggesting a genuine dilution into the larger tuber mass, Zn appeared to maintain its concentration through mechanisms we attribute to the progressive build‐up of Zn‐solubilizing microbial communities and organically‐complexed soil Zn across the two cropping cycles. This stability is agronomically valuable: it means that higher‐yielding OFSP plants under OWP + MIs management do not ‘dilute’ their Zn content, so consumers benefit from both larger quantities and maintained mineral density per unit weight. The contrast with the dilution reported for Zn in high‐biomass sugarcane (Pouzet et al. 1999) suggests that the biological Zn mobilization sustained by OWP + BM inputs successfully compensated for the increased plant sink demand in Season 2.

Compared to Fe, Zn shows a more balanced input‐extraction budget: OFSP tubers extracted a proportion of Zn exceeding the amounts applied via PL (308% extraction rate, Table S5), indicating that the crop drew on pre‐existing soil Zn reserves in addition to freshly supplied inputs. SS, however, still left a net positive Zn residue (0.69 kg Zn ha^−1^, Table S5). Across multiple OFSP cropping cycles with SS, this residual Zn could accumulate to phytotoxic thresholds (> 500 ppm), compromising both OFSP root health and the activity of the BM community—which itself constitutes a key element of the system's long‐term productivity (Hasnine et al. 2017). Periodic soil Zn monitoring and dose adjustment between SS applications are therefore warranted in this production system.

### 4.4 Implications for Fe and Zn Bioavailability

Beyond the quantitative gains in total mineral concentration, a key question is whether the Fe (up to +69%) and Zn (up to +100%, Figure 3) increases observed in OFSP tubers under the most effective treatments are likely to translate into improved bioavailability for target populations. Several converging lines of evidence suggest they are. Huey et al. (2024) demonstrated that biofortified crops consistently exhibit higher Fe and Zn bioaccessibility than their non‐biofortified counterparts, indicating that increased total mineral concentration achieved through agronomic management tends to co‐occur with, rather than oppose, improved intestinal absorption. More directly, Thiam, Diouf, Icard‐Vernière, et al. (2025) showed within the same OR4Food experimental framework—using the same OWPs and BMs under the same pedoclimatic conditions of Senegal's Peanut Basin—that PL combined with MIs increased in vitro Fe bioaccessibility in cowpea from 9% (control) to 27%–29%, and that β‐carotene bioaccessibility in OFSP reached 4.1% compared to 1.4%–2.5% in non‐biofortified samples. Although these data were generated on different crops and plant organs, they constitute the most directly transferable proxy evidence available for our study system, and strongly suggest that the mineral gains reported here carry tangible nutritional relevance.

A more complete assessment of bioavailability would, however, require quantifying phytic acid content in the biofortified tubers. As the predominant antinutritional factor in sweet potato, phytic acid forms insoluble complexes with Fe^2+^ and Zn^2+^ that can limit their intestinal absorption (Koua et al. 2018), and its concentration may vary across treatments depending on the phosphorus supply from OWPs. While phytate quantification and in vitro bioaccessibility estimations using standardized protocols (such as INFOGEST digestion or the Hill method) fell outside the initial operational scope of this agronomic field study, these analyses represent a critical next step and are identified as a priority for future work.

## 5 Conclusion

In several cases, the combined application of OWPs and microbial inocula significantly increased Fe and Zn concentrations in OFSP tubers. These results confirm our initial hypotheses, namely: (1) the potential for leveraging the micronutrients contained in OWPs for the biofortification of OFSP within an agroecological context; and (2) the agronomic value of incorporating microbial inocula to maximize micronutrient gains. Accordingly, the combination of PL (2.05 t ha^−1^), BMs (9.44 t ha^−1^), and MF (1.88 t ha^−1^) can be recommended for the agro‐biofortification of Fe and Zn in OFSP tubers. Future studies should include (i) an assessment of the bioaccessible fractions of Fe and Zn in agrobiofortified OFSP tubers using standardized in vitro digestion protocols, such as INFOGEST or the Hill method, to determine whether the observed increases in mineral concentration translate into meaningful nutritional benefits; (ii) characterization of soil microbial community dynamics using PLFA or metabarcoding approaches (16S rRNA and ITS), in order to identify the functional microbial groups involved in Fe and Zn mobilization in the rhizosphere during the growing season and to determine how OWP × microbial inocula combinations reshape the soil microbiome; (iii) multi‐site and multi‐variety validation through field trials conducted in contrasting agroclimatic zones of Senegal and using diverse OFSP genetic material, to verify the generalizability of the optimal treatment combinations identified in this study; and (iv) long‐term monitoring of Fe and Zn partitioning in soil to evaluate potential toxicity risks and nutrient imbalances across successive crop cycles.

## Author Contributions

**Emmanuel Noumsi‐Foamouhoue:** conceptualization, methodology, software, data curation, investigation, validation, formal analysis, visualization, writing – original draft, writing – review and editing. **Paula Fernandes:** conceptualization, methodology, investigation, validation, supervision, funding acquisition, project administration, resources, writing – review and editing. **Samuel Legros:** conceptualization, methodology, software, investigation, validation, formal analysis, supervision, visualization, project administration, writing – review and editing. **Hassna Founoune‐Mboup:** conceptualization, methodology, investigation, validation, resources, writing – review and editing. **Bassirou Diallo:** methodology, validation, resources, writing – review and editing. **Komi Assigbetsé:** methodology, investigation, validation, writing – review and editing. **Aboubacry Kane:** methodology, investigation, validation, supervision, project administration, writing – review and editing. **Frédéric Feder:** methodology, investigation, validation, supervision, project administration, writing – review and editing. **Jean‐Michel Médoc:** conceptualization, methodology, investigation, validation, supervision, funding acquisition, project administration, writing – review and editing, resources.

## Funding

This research was funded by African Union Commission and European Union Commission under grant number AURG‐II‐2‐110‐2018. Additional support for the valorization and dissemination of results was provided by the World Bank through the NBS Invest Fund (Accelerating Nature‐Based Solutions in Least Developed Countries), within the framework of the ASA Reflection and Knowledge project (contract number 0002010826).

## Ethics Statement

The authors have nothing to report.

## Consent

The authors have nothing to report.

## Conflicts of Interest

The authors declare no conflicts of interest.

## Supporting information


**Table S1:** Diversity and abundance of local beneficial microorganisms (Noumsi‐Foamouhoue et al., 2023).
**Table S2:** Quantities of N, P, and K supplied by OWP and chemical supplements.
**Table S3:** Quantities of iron and zinc provided by OWP and BM.
**Table S4:** Effects of seasons, treatments, and seasons × treatments interaction on orange‐fleshed sweet potato yields, Fe and Zn concentrations in tubers, and Fe and Zn yields in tubers.
**Table S5:** Iron and zinc extraction rates by orange‐fleshed sweet potato tubers.
**Figure S1:** Meteorological data from the Institut Sénégalais de Recherches Agricoles (ISRA) experimental station (data source. SDDR, 2022).
**Image S1:** Total orange‐fleshed sweet potato plot after weeding, Season 1, ISRA experimental station, Nioro du Rip, Senegal.
**Image S2:** Harvesting of orange‐fleshed sweet potato, Season 1, ISRA experimental station, Nioro du Rip, Senegal. a, digging up mature tubers; b, heap of harvested tubers; c, storage of tubers in harvest crates.

## Data Availability

Data will be made available on request.
