# Supplementary material for: Organic‐Microbial Strategies for Iron and Zinc Biofortification in Orange‐Fleshed Sweet Potato [ Ipomoea batatas (L.) Lam.]
Source: Food Sci Nutr. 2026 Sep 3;14(9):e72323. doi: 10.1002/fsn3.72323 (PMC13539350; doi:10.1002/fsn3.72323)
Supplement: Supplementary file 1 [file FSN3-14-e72323-s001.docx]

**Supplementary Material**

**Organic-Microbial Strategies for Iron and Zinc Biofortification in Orange-Fleshed Sweet Potato [*Ipomoea batatas* (L.) Lam.]**

Emmanuel Noumsi-Foamouhoue^1,2,3*^, Paula Fernandes^2,4,5^, Samuel Legros^1,3,6^, Hassna Founoune-Mboup^8^, Bassirou Diallo^8^, Komi Assigbetsé^2,7^, Aboubacry Kane^9,10^, Frédéric Feder^1,3^, Jean-Michel Médoc^1,2,3*^

1 CIRAD, UPR Recyclage et Risque, F-34398 Montpellier, France

2 Laboratoire Mixte International IESOL, ISRA-IRD Bel-Air Center, Dakar BP 1386, Senegal

3 Recyclage et Risque, Université de Montpellier, Montpellier, France

4 UPR HortSys, CIRAD, F-34398 Montpellier, France

5 HortSys, Université de Montpellier, Montpellier, France

6 CIRAD, UPR Recyclage et Risque, Saint-Denis, La Réunion F-97743, France

7 Institut de Recherche pour le Développement (IRD), UMR Eco&Sols F-34394 Montpellier, France

8 Laboratoire National de Recherches sur les Productions Végétales (LNRPV), Institut Sénégalais de Recherche Agricole (ISRA), Dakar, Senegal

9 FST, Département de Biologie Végétale, UCAD, Dakar BP 1386, Senegal

10 Laboratoire Commun de Microbiologie (LCM), IRD-ISRA-UCAD Bel-Air Center, Dakar BP 1386, Senegal

***Correspondence**: emmanuelnofoma@yahoo.com or [emmanuel.noumsi_foamouhoue@cirad.fr](mailto:emmanuel.noumsi_foamouhoue@cirad.fr) (ENF); [jean-michel.medoc@cirad.fr](mailto:jean-michel.medoc@cirad.fr) (JMM)

**Supplementary Tables**

**Table S1**

Diversity and abundance of local beneficial microorganisms (Noumsi-Foamouhoue et al., 2023).

|  | Groups | Genus | Relative Abundance |
| --- | --- | --- | --- |
| Bacteria  (% OTU) | *Proteobacteria* | *Acetobacter* | 28.45 |
|  |  | *Acinetobacter* | 0.01 |
|  |  | *Pseudomonas* | 0.01 |
|  |  | *Enterobacter* | 0.34 |
|  |  | *Pantoea* | 0.13 |
|  |  | *Rhizobium* | 0.05 |
|  |  | *Methylobacterium* | 0.01 |
|  |  | *Stenotrophomonas* | 0.01 |
|  |  | *Burkholderia* | 0.01 |
|  |  | *Bradyrhizobium* | 0.03 |
|  |  | *Mesorhizobium* | 0.00 |
|  |  | *Devosia* | 0.03 |
|  |  | *Pseudoaminobacter* | 0.00 |
|  | *Actinobacteria* | *Mycobacterium* | 0.01 |
|  | *Firmicutes* | *Lactobacillus* | 40.00 |
|  |  | *Bacillus* | 5.35 |
|  |  | *Weissella* | 5.08 |
|  |  | *Paenibacillus* | 0.27 |
|  |  | *Clostridium* | 8.01 |
|  |  | *Lysinibacillus* | 0.83 |
|  |  | *Rummeliibacillus* | 2.92 |
|  |  | *Pediococcus* | 0.74 |
|  |  | *Virgibacillus* | 0.01 |
|  | *Bacteroidota* | *Sphingobacterium* | 0.02 |
|  |  | *Chryseobacterium* | 0.00 |
| Fungi  (% OTU) | *Ascomycota* | *Neoascochyta* | 33.75 |
|  |  | *Ascochyta* | 3.59 |
|  |  | *Myrothecium* | 2.13 |
|  |  | *Cladosporium* | 0.01 |
|  |  | *Colletotrichum* | 0.05 |
|  | Basidiomycota | *Yueomyces* | 2.11 |

OTU (operational taxonomic units).

**Table S2**

Quantities of N, P, and K supplied by OWP and chemical supplements.

|  | | | N | P | K | Calculation | References |
| --- | --- | --- | --- | --- | --- | --- | --- |
| Recommended dose of nutrient (kg ha^−1^) | | | 80 | 65.49 | 132.48 | (1) | (Gret et al., 2002) |
| Fertilizer equivalence coefficient | PL | | 0.6 | 0.65 | 1 | (2) | (Leclerc, 2001) |
|  | SS | | 0.45 | 0.6 | 1 |  |  |
| Total concentration of the element (kg kg^−1^ DM) | PL | | 0.065 | 0.012 | 0.032 | (3) |  |
|  | SS | | 0.025 | 0.009 | 0.002 |  |  |
| Effective concentration of the element (kg kg^−1^ DM) | PL | | 0.039 | 0.008 | 0.032 | (4) = (3) × (2) |  |
|  | SS | | 0.011 | 0.005 | 0.002 |  |  |
| Theoretical quantity of OWP (t DM ha^−1^) | PL | | 2.05 | 8.19 | 4,14 | (5) = (1) ÷ [1000 × (4)] |  |
|  | SS | | 7.27 | 13.1 | 66.24 |  |  |
| Actual quantity of OWP (t DM ha^−1^) | PL | 2.05 |  | | | (6) |  |
|  | SS | 7.27 |  | | |  |  |
| Actual quantity of element supplied with OWP (kg ha^−1^) | PL | | 79.95 | 16.4 | 65.6 | (7) = (4) × [1000 × (6)] |  |
|  | SS | | 79.97 | 36.35 | 14.54 |  |  |
| Quantity of element supplied with mineral supplements (kg ha^−1^) | PL | | 0 | 49.1 | 66.88 | (8) = [(5 − 6) × (1)] ÷ (5) |  |
|  | SS | | 0 | 29.15 | 117.94 |  |  |
| Total quantity of element supplied (kg ha^−1^) | PL | | 79.95 | 65.5 | 132.48 | (7) + (8) |  |
|  | SS | | 79.97 | 65.5 | 132.48 |  |  |

OWP (organic waste product), PL (poultry litter), SS (sewage sludge), N (nitrogen), P (phosphorus), K (potassium), DM (Dry Matter).

**Table S3**

Quantities of iron and zinc provided by OWP and BM.

|  |  | | Fe | Zn | Calculation |
| --- | --- | --- | --- | --- | --- |
| Total concentration of the element (mg kg^−1^ MS) | PL | | 6210 | 326 | (1) |
|  | SS | | 15724 | 364 |  |
|  | BM | | 4.34 | 20 |  |
| Quantity of OWP and BM applied (t MS ha^−1^) | PL | 2.05 |  | | (2) |
|  | SS | 7.27 |  | |  |
|  | BM | 9.44 |  | |  |
| Total concentration of the element (kg ha^−1^) | PL | | 12.73 | 0.67 | (3) = [(1) × (2)] ÷ 1000 |
|  | SS | | 114.31 | 2.65 |  |
|  | BM | | 0.04 | 0.2 |  |

OWP (organic waste product), PL (poultry litter), SS (sewage sludge), BM (local beneficial microorganisms), Fe (iron), Zn (zinc), DM (Dry Matter).

**Table S4**

Effects of seasons, treatments, and seasons x treatments interaction on orange-fleshed sweet potato yields, Fe and Zn concentrations in tubers, and Fe and Zn yields in tubers.

|  | **Variation source** | **Modalities** | **Mean ± SD (t ha^−1^)** | **F** | **Pr** |
| --- | --- | --- | --- | --- | --- |
| **Effects on OFSP yields** | Seasons | Season 1 | 10.05 ± 2.63^b^ | 124 | < 0.001 |
|  |  | Season 2 | 15.87 ± 4.68^a^ |  |  |
|  | Treatments |  |  | 13.67 | < 0.001 |
|  | Seasons × treatments |  |  | 2.52 | < 0.05 |
| **Effects on Fe concentration in tubers** | Seasons | Season 1 | 44 ± 9.53^a^ | 50.83 | < 0.001 |
|  |  | Season 2 | 30 ± 10.94^b^ |  |  |
|  | Treatments |  |  | 3.84 | < 0.001 |
|  | Seasons × treatments |  |  | 1.76 | 0.096 |
| **Effects on Zn concentration in tubers** | Seasons | Season 1 | 12.82 ± 2.54 | 2.65 | 0.112 |
|  |  | Season 2 | 13.83 ± 3.98 |  |  |
|  | Treatments |  |  | 3.85 | < 0.01 |
|  | Seasons × treatments |  |  | 5.12 | < 0.001 |
| **Effects on Fe yields in tubers** | Seasons | Season 1 | 444 ± 164 | 2.82 | 0.098 |
|  |  | Season 2 | 490 ± 242 |  |  |
|  | Treatments |  |  | 14.40 | < 0.001 |
|  | Seasons × treatments |  |  | 3.49 | < 0.01 |
| **Effects on Zn yields in tubers** | Seasons | Season 1 | 120 ± 40^b^ | 58.85 | < 0.001 |
|  |  | Season 2 | 226 ± 90^a^ |  |  |
|  | Treatments |  |  | 4.75 | < 0.001 |
|  | Seasons × treatments |  |  | 2.39 | < 0.05 |

**Table S5**

Iron and zinc extraction rates by orange-fleshed sweet potato tubers.

|  | |  | Supplied quantities (kg/ha) | Extracted quantities (kg/ha) | Extraction rate (%) | Residual quantities in soil (kg/ha) |
| --- | --- | --- | --- | --- | --- | --- |
|  |  |  |  |  |  |  |
| Iron | PL | Season 1 | 12.73 | 8.01 | 62.92 | 4.72 |
|  |  | Season 2 | 12.73 | 9.75 | 76.59 | 2.98 |
|  |  | Sum | 25.46 | 17.76 | 69.76 | 7.7 |
|  | SS | Season 1 | 114.31 | 7.63 | 6.67 | 106.68 |
|  |  | Season 2 | 114.31 | 8.21 | 7.18 | 106.1 |
|  |  | Sum | 228.62 | 15.84 | 6.93 | 212.78 |
| Zinc | PL | Season 1 | 0.67 | 1.42 | 211.94 | −0.75 |
|  |  | Season 2 | 0.67 | 2.71 | 404.48 | −2.04 |
|  |  | Sum | 1.34 | 4.13 | 308.21 | −2.79 |
|  | SS | Season 1 | 2.65 | 1.24 | 46.79 | 1.41 |
|  |  | Season 2 | 2.65 | 3.37 | 127.17 | −0.72 |
|  |  | Sum | 5.3 | 4.61 | 86.98 | 0.69 |

PL (poultry litter), SS (sewage sludge).

Supplementary Figure

**Figure S1**

**Figure S1.** Meteorological data from the Institut Sénégalais de Recherches Agricoles (ISRA) experimental station (data source. SDDR, 2022).

Supplementary images

**Image S1**

**Image S1.** Total orange-fleshed sweet potato plot after weeding, season 1, ISRA experimental station, Nioro du Rip, Senegal.

**Image S2**

**Image S2.** Harvesting of orange-fleshed sweet potato, season 1, ISRA experimental station, Nioro du Rip, Senegal. a = digging up mature tubers; b = heap of harvested tubers; c = storage of tubers in harvest crates.

**References**

Gret, Cirad, & Ministère des Affaires étrangères (2003). *Mémento de l’agronome* (4ᵉ éd.). Paris, France: Gret / Cirad. ISBN 978-2-86844-129-7 - 978-2-87614-522-7

Leclerc, B. (2001). *Guide des matières organiques* Institut Technique de l’Agriculture Biologique & C. Paris (Eds.), *2* p. 12.

Noumsi-Foamouhoue, E., Legros, S., Fernandes, P., Thuriès, L., Assigbetsé, K., Kane, A., . . . Médoc, J.-M. (2023). Local beneficial microorganisms impact carbon and nitrogen mineralization in a lixisol incubated with organic waste products. *Agronomy*, *13*(11), 2791. doi:10.3390/agronomy13112791

SDDR. (2022). *Services départementaux de développement rural*. Nioro du Rip, Sénégal.
